# Profiling of 5-hydroxymethylcytosine in blood reveals preferential enrichment at exon-intron junctions and predictive value for Parkinson’s disease

**DOI:** 10.1038/s41531-026-01322-x

**Published:** 2026-03-20

**Authors:** Philipp Antczak, Peter Brandt, Lav Radosavljević, Per Svenningsson, Joëlle Rüegg, Kristina Bečanović

**Affiliations:** 1 Department II of Internal Medicine, University of Cologne, Faculty of Medicine and University Hospital Cologne, Cologne, Germany; 2 Center for Molecular Medicine Cologne, University of Cologne, Faculty of Medicine and University Hospital Cologne, Cologne, Germany; 3 Cologne Excellence Cluster on Cellular Stress Responses in Aging-Associated Diseases (CECAD), Cologne, Germany; 4 Department of Medicinal Chemistry, Uppsala University, Uppsala, Sweden; 5 Nuffield Department of Population Health, University of Oxford, Oxford, United Kingdom; 6 Department of Clinical Neuroscience, Karolinska Institutet, Stockholm, Sweden; 7 Department of Organismal Biology, Uppsala University, Uppsala, Sweden; 8 Present Address: Beactica Therapeutics AB, Uppsala, Sweden

**Keywords:** Biomarkers, Diseases, Genetics, Neurology, Neuroscience

## Abstract

5-methylcytosine (5mC) and 5-hydroxymethylcytosine (5hmC) are epigenetic modifications increasingly recognized for their roles in Parkinson’s disease (PD). In this study, we profiled 5mC and 5hmC in blood samples from individuals with PD to explore their relevance to disease status and progression. We observed significantly reduced global 5hmC levels in peripheral blood mononuclear cells (PBMCs) from PD cases compared to controls. Using the Illumina EPIC BeadArray, we conducted genome-wide analyses of 5mC and 5hmC in a subset of PD cases and controls to explore methylation and hydroxymethylation patterns. We identified differentially methylated and hydroxymethylated positions and regions enriched within introns, with both types of regions showing a marked concentration near exon-intron junctions. Positional analysis relative to exon–intron junctions revealed that proximal and distal regions mapped to partially different functional themes, suggesting that genomic context provides additional biological insight. Functional enrichment analyses also highlighted distinct biological roles for 5mC and 5hmC, with associated genes implicated in neurodevelopment, vascular remodeling, and neuroimmune signaling. Additionally, we demonstrate that global 5hmC levels, in combination with age and sex, are predictive of disease status, highlighting the potential of 5hmC as a blood-based biomarker for PD.

## Introduction

Parkinson’s disease (PD) is a progressive neurodegenerative disorder characterized by the degeneration of dopaminergic neurons in the substantia nigra pars compacta (SNc) and the accumulation of intracellular inclusions containing aggregates of *α*-synuclein^1^. Clinically, PD presents with motor symptoms such as resting tremor, rigidity, bradykinesia, and postural instability, as well as non-motor symptoms including constipation, depression, sleep disorders, and cognitive dysfunction. The underlying molecular pathogenesis involves multiple mechanisms and pathways, including α-synuclein proteostasis, mitochondrial function, oxidative stress, calcium homeostasis, axonal transport, and neuroinflammation^2^. While the majority (90%) of PD cases are idiopathic, both environmental and genetic factors contribute to disease manifestation^3^. A recent meta-analysis of genome-wide association studies identified novel disease risk loci explaining 16–36% of the heritable risk of PD, suggesting that a significant genetic component remains unidentified^4^. Epidemiological studies indicate protective effects of smoking, alcohol, and caffeine consumption, whereas exposure to pesticides, solvents, heavy metals, and traumatic brain injury is linked to increased PD risk^1,5^. PD prevalence is higher in men than in women and increases with age, affecting approximately 1% of individuals at 65 years, and rising to 4–5% by 85-year-old^1^. Given the multifactorial nature of PD, epigenetics may provide a critical link between genetic and environmental influences in idiopathic PD. Several features of PD, such as discordance in monozygotic (MZ) twins, sexual dimorphism, and age-related pathology, are now being explained in part through epigenetic modifications^6^.

Epigenetic mechanisms, including DNA methylation, histone modification, and noncoding RNA-associated gene modulation, regulate gene expression, cellular differentiation, and development of tissues, including brain development^7^. DNA methylation, catalyzed by DNA methyltransferases, leads to the formation of 5-methylcytosine (5mC), a crucial regulator of gene expression^8^. Disruptions in this system contribute to disease pathology and globally altered DNA methylation patterns^9^. Additionally, DNA hydroxymethylation represents a more recently identified epigenetic modification, where ten-eleven translocation (TET) proteins oxidize 5mC to produce 5-hydroxymethylcytosine (5hmC) as a first step in the DNA demethylation process^10,11^. 5hmC levels vary across tissues, with the highest levels found in the adult brain (between <0.1 and 0.7%) but lower and more variable than 5mC levels (4–5% of all cytosines). 5hmC and TET proteins also play critical roles in epigenetic reprogramming and tissue-specific gene regulation^12,13^.

Previous studies have reported altered global methylation and hydroxymethylation levels in PD *postmortem* cortex and cerebellum samples^14,15^. Genome-wide analyses further revealed differential methylation and hydroxymethylation at enhancers in prefrontal cortex neurons of individuals with PD^16^. More recently, differentially hydroxymethylated regions (DhMRs), but not differentially methylated regions (DMRs), were identified in the substantia nigra of PD cases^17^. However, brain tissue studies do not allow for longitudinal disease progression analysis or assessment of therapeutic interventions^18^, necessitating investigations in more accessible tissues such as blood. Indeed, genome-wide methylation changes in *postmortem* brain samples have been found to be concordant with those in peripheral blood mononuclear cells (PBMCs) of individuals with PD, supporting the use of blood as a surrogate tissue for studying epigenetic alterations^19^. Epigenetic variations in specific genes, measured in PBMCs of discordant siblings and monozygotic twins, have been associated with PD susceptibility^20^. Moreover, recent findings indicate reduced global 5mC and 5hmC levels in buffy coat samples from individuals with PD and Alzheimer’s disease (AD)^21^.

In this study, we investigated global and locus-specific DNA methylation and hydroxymethylation patterns in PBMCs to determine whether epigenetic alterations are associated with PD status and clinical progression. By combining global 5hmC quantification in a large cohort with exploratory genome-wide profiling in a subset of samples, we aimed to identify disease-related epigenetic signatures and evaluate their potential relevance for biomarker development. This integrated approach addresses a major gap in the current PD epigenomics literature, where 5hmC remains largely unexplored.

## Results

### Global hypo-hydroxymethylation in PBMCs of PD patients

**Fig. 1:**
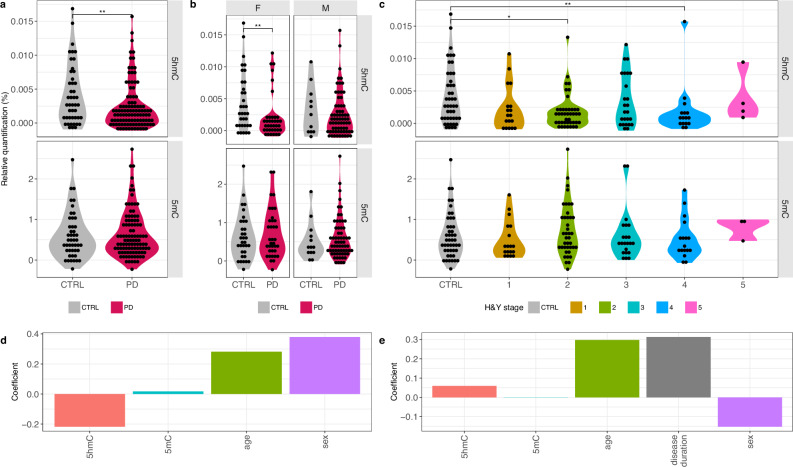
Global 5mC and 5hmC levels of peripheral blood mononuclear cells (PBMCs) from PD and control (CTRL) subjects. **a** Global 5hmC (upper panel) and 5mC (lower panel) levels in PD and CTRL subjects. **b** Global 5mC and 5hmC levels stratified by sex. **c** Global 5mC and 5hmC levels across different Hoehn & Yahr stages (H&Y). **d** Partial least squares discriminant analysis (PLS-DA) comparing PD and CTRL groups using global 5mC and 5hmC levels, sex (female = 0; male = 1), and age as descriptor variables (*x*-axis). **e** PLS analysis predicting H&Y stages using sex, age, disease duration, and global levels of 5mC and 5hmC as descriptor variables (*x*-axis). The *y*-axis represents scaled and centered regression coefficients in (**d**, **e**). F-tests, adjusted for sex and age, were used to compare PD and CTRL subjects in (**a**–**c**). Global DNA methylation/hydroxymethylation data are reported as mean ± SEM based on raw values. Box and whisker plots show the median, with boxes spanning the 25-75th percentiles, and whiskers extending to the minimum and maximum values. *P < 0.05, **P < 0.01.

To examine global 5mC and 5hmC levels in PBMCs of individuals with PD and control subjects, we employed an ELISA-based approach. Flow-cytometry data previously generated for a representative subset of this cohort showed no significant differences in major PBMC subsets between PD and control groups^22^, indicating that global methylation differences are unlikely to reflect shifts in leukocyte composition. The degree of motor impairment and disability in individuals with PD was determined using the Hoehn and Yahr (H&Y) scale (Table 1). Our findings showed interindividual variability in global 5mC and 5hmC levels in both PD and control samples (Fig. 1a). Notably, global 5hmC levels were significantly lower in PD cases (mean ± SEM 5hmC 0.0024% ± 0.0003) compared to controls (mean ± SEM 5hmC 0.0041% ± 0.0007) (*p* = 0.0045) (Fig. 1a). Stratification by sex showed significantly lower global 5hmC levels in female PD cases (mean 5hmC 0.0022% ± 0.0006) compared to female controls (mean 5hmC 0.0045% ± 0.0008) (Fig. 1b) (*p* = 0.0058). However, this difference was not observed in male PD cases (mean 5hmC 0.0025% ± 0.0004) compared to male controls (mean 5hmC 0.0032% ± 0.0001) (*p* = 0.398), which may be due to the low number of male control samples (Table 1). When analyzing disease stages, PD cases at H&Y stages 2 (mean 5hmC 0.0018% ± 0.0004) (*p* = 0.007) and 4 (mean 5hmC 0.0019% ± 0.001) (*p* = 0.020) exhibited significantly lower 5hmC levels compared to controls (mean 5hmC 0.0041% ± 0.0007) (Fig. 1c). In contrast, global 5mC levels did not differ significantly between PD cases (mean 5mC 0.63% ± 0.058) and controls (mean 5mC 0.601% ± 0.084) (*p* = 0.78) (Fig. 1a), even when data was stratified by sex (Fig. 1b) or disease stages (Fig. 1c).

**Table 1:** Demographic and clinical characteristics of study participants

Group	H&Y stage^a^	Number (n)^b^	Sex M/F (n)	Age at blood sampling^c^ mean±SD	Age at diagnosis mean±SD	Disease duration^d^ (years) mean± SD
PD	1	19	15/4	58.8 ± 11.5	57.1 ± 11.5	1.8 ± 3.0
	2	44	32/12	69.0 ± 6.8	63.0 ± 7.5	6.1 ± 4.9
	3	25	14/11	69.2 ± 11.2	59.8 ± 11.1	9.4 ± 5.2
	4	17	9/8	74.8 ± 8.5	66.6 ± 11.2	8.1 ± 6.2
	5	4	2/2	72.8 ± 6.8	61.3 ± 5.9	11.5 ± 4.5
All PD Controls		109 49	72/37 13/36	68.3 ± 10.2 62.0 ± 11.0	61.7 ± 10.0 NA^e^	6.6 ± 5.5 NA^d^

^a^Hoehn & Yahr stage at blood sampling/PBMC isolation.

^b^Number of GBA E326K carriers: H&Y stage 2 (*n* = 5), H&Y stage 3 (*n* = 2), H&Y stage 4 (*n* = 2).

^c^Age at blood sampling/PBMC isolation.

^d^Disease duration equals years between clinical PD diagnosis and blood sampling/PBMC isolation.

^e^NA = not applicable.

Genetic studies have implicated the GBA E326K variant as a potential PD risk factor^23,24^. To account for this, we excluded nine carriers in our cohort from the analysis, and the results remained consistent, with PD cases (mean ± SEM 5hmC 0.0022% ± 0.0003) exhibiting significantly lower 5hmC levels than controls (mean 5hmC 0.0041% ± 0.0007) (*p* = 0.0045).

We conducted a multivariate analysis using partial least squares discriminant analysis (PLS-DA), a supervised classification method that identifies the variables that best distinguish between groups. Sex, age, and global 5mC and 5hmC levels were included as descriptor variables to evaluate their combined ability to separate PD cases from controls. The analysis yielded one significant principal component (PC) explaining 26% of the variance (*Q*^2^ = 22%). Regression coefficients (*B*) indicated a positive association between PD and sex (male) (*B*_sex_ *=* 0.38 ± 0.23; VIP = 1.49) and age (*B*_age_ = 0.28 ± 0.10; VIP = 0.96), whereas global 5hmC levels showed a negative association (*B*_5hmC_ = −0.22 ± 0.22; VIP = 0.92) (Fig. 1d). Global 5mC levels were unimportant for the model (*B*_5mC_ = 0.02 ± 0.20; VIP = 0.15). We then applied further PLS regression analyses, a supervised multivariate method suitable for analyzing high-dimensional collinear data, to explore associations between H&Y stage and sex, age, disease duration, and global 5mC and 5hmC levels. One significant PC explained 29% of the variance (*Q*^2^ = 23%). As expected, given the progressive and irreversible nature of PD, age (*B*_age_ = 0.30 ± 0.14; VIP = 1.44) and disease duration (*B*_duration_ = 0.31 ± 0.28; VIP = 1.52) were positively associated with disease severity, while 5hmC levels (*B*_5hmC_ = 0.06 ± 0.15; VIP = 0.29), 5mC levels (*B*_5mC_ = 0.0009 ± 0.17; VIP = 0.005) and sex (*B*_sex_ = −0.15 ± 0.16; VIP = 0.74) were less influential (Fig. 1e). To assess whether medication use and other exposures contributed to variation in global 5mC and 5hmC levels, we performed PLS analyses including medication variables together with sex, age, smoking, exposure to pesticides, heavy metals or paint/solvents, and caffeine intake as X-variables. Three medication categorizations were evaluated: individual PD-specific drugs for motor symptom treatment (*n* = 12), PD-specific drugs grouped by mechanism of action (seven categories) (Table S1), and dopamine replacement classified as “intake” versus “no intake”. Among these analyses, only the dataset using drugs grouped by mechanism of action produced a model, albeit with no predictive ability (*Q*² = −0.02 and failing the permutation test). Exploratory examination of VIP scores showed that exposure to paints/solvents (VIP = 2.70) and caffeine intake (VIP = 2.09) contributed more strongly to the component than medication variables (all VIP < 0.87). Overall, these analyses indicate that medication status did not substantially influence global 5mC or 5hmC levels. A direct comparison between levodopa-treated (*n* = 77; mean 0.0022% ± 0.0004) and untreated (*n* = 28; mean 0.0027% ± 0.0008) individuals with PD also showed no significant difference in 5hmC (*p* = 0.52) or in 5mC levels between levodopa-treated (*n* = 73; mean 0.66% ± 0.074) and untreated individuals with PD (*n* = 27; mean 0.54% ± 0.074) (*p* = 0.38).

In summary, we identified a significant association between reduced global 5hmC levels and PD status, but not disease stage. This reduction was independent of medication intake and known risk or protective factors for PD.

### Genome-wide mapping of 5mC and 5hmC alterations in PBMCs reveals intronic enrichment and CpG island depletion

To better understand how 5mC and 5hmC patterns are altered in the context of PD, we compared methylation states at individual CpG sites between individuals at H&Y stage 4 and matched controls (Table S2). This analysis identified 92 differentially methylated positions (DMPs) and 35 differentially hydroxymethylated positions (DhMPs) at a family-wise error rate (FWER) < 0.05 (Data S1). Significant DMPs showed both hypermethylation (50/92, 54.3%) and hypomethylation (42/92, 45.7%), whereas DhMPs were predominantly hypomethylated (25/35, 71.4%). Given the small sample size, p-values and effect estimates at individual CpGs should be interpreted cautiously, as both false positives and false negatives are possible. For this reason, we focus primarily on the direction of change rather than the magnitude of test statistics.

**Fig. 2:**
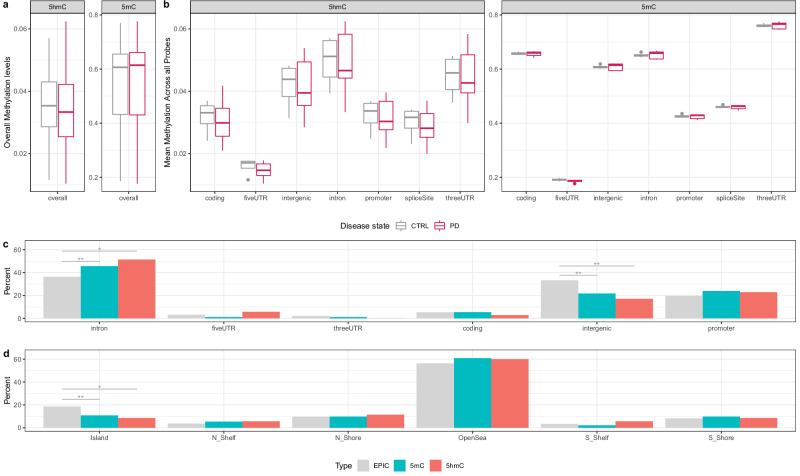
Genome-wide analysis of 5mC and 5hmC in PD patients (Hoehn & Yahr stage 4) and controls (CTRL). **a** Genome-wide 5mC and 5hmC profiling assessed using the Illumina EPIC beadarray. **b** Mean methylation levels across different genomic features for all genome-wide 5hmC probes (left panel) and 5mC probes (right panel). **c**, **d** Percent enrichment of significant differentially methylated (DMPs) and hydroxymethylated positions (DhMPs) across genomic features and CpG island annotations. Box and whisker plots show the median, with boxes spanning the 25-75th percentiles, and whiskers extending to the minimum and maximum values. Bars represent the percentage change in observed versus expected counts for each category, based on genome-wide distribution. Intronic regions showed significant enrichment for both DMPs and DhMPs, while intergenic regions and CpG islands were significantly depleted compared to the full array. *P < 0.1, **P < 0.05.

Next, we assessed the genomic distribution of DMPs and DhMPs. CpG sites located near exons were more frequently differentially modified between PD cases and controls, suggesting that proximity to exons may increase susceptibility to methylation changes (Fig. S1). Although genome-wide profiling revealed no differences in overall mean 5mC and 5hmC levels between groups (Fig. 2a), intronic regions exhibited higher mean 5mC and 5hmC levels across different genomic regions (Fig. 2b). To evaluate whether disease-associated changes were preferentially localized to introns, we performed a bootstrapping analysis across the full array. This analysis demonstrated significant enrichment of DMPs (*p* = 0.030) and DhMPs (*p* = 0.026) within intronic regions compared to other genomic contexts (Fig. 2c), whereas intergenic regions were significantly depleted for both modifications (DMPs *p* = 0.013; DhMPs *p* = 0.030) (Fig. 2c). Additionally, we observed a depletion of CpG island-associated probes among DMPs and DhMPs, with both 5mC (*p* = 0.036) and 5hmC (*p* = 0.087) showing statistical significance at an false discovery rate (FDR) < 0.1 (Fig. 2d). Chromosomal mapping of D(h)MPs and D(h)MRs did not reveal any specific regional clustering (Fig. S2). Collectively, these findings suggest that D(h)MPs are enriched in intronic regions compared to other genomic contexts, raising the possibility that intronic regions may be preferentially affected by epigenetic changes in PD.

### D(h)MPs are linked to neuronal and developmental processes in PD

To assess the biological relevance of the identified D(h)MPs, we performed functional enrichment analysis on genes associated with the DMPs, excluding intergenic regions. DMP-associated genes showed significant enrichment in neurodevelopmental and synaptic processes. Notably, *TNIK, BAIAP2, GRIK5, KCNQ1, OLFM1, PLXNA4, CDH4*, and *PTPRQ* were linked to pathways related to neuron differentiation, axon extension, membrane repolarization, and synaptic signaling (Fig. S3, Data S2). These genes were also associated with structural components such as the postsynaptic density, ion channel complexes, and neuron projections. In contrast, DhMP-associated genes were predominantly enriched in developmental and sensory pathways. Key genes included *SMARCA4, HIPK2, ZHX2*, and *AHI1*, which contributed to broader categories such as system development, cell differentiation, and enzyme-linked receptor signaling (Fig. S3, Data S2).

### DMRs and D(h)MRs reveal forebrain development, vasculogenesis, and signal transduction pathway enrichment in PD

Given the clustered nature of DNA methylation, we sought to identify differentially methylated regions (DMRs) and differentially hydroxymethylated regions (DhMRs) within the 5mC and 5hmC datasets, respectively, by comparing PD cases to controls. At a stringent FWER threshold of <5%, we identified nine significant DhMRs, while no DMRs passed this threshold. To enable exploratory, hypothesis-generating analyses, we relaxed the threshold to FWER ≤ 20%, resulting in the identification of 18 DMRs and 107 DhMRs (Data S1). Notably, 77 out of 107 (72%) DhMRs were located within annotated genes, compared to 9 of the 18 (50%) DMRs (Data S1). DMRs showed a mixture of hyper- and hypomethylated regions. Probe-level statistics within each region generally supported the regional direction, although individual CpGs did not always shift uniformly. In contrast, DhMRs were predominantly hypohydroxymethylated (96/107, 89.7%), with a smaller number of DhMRs exhibiting increased 5hmC (11/107, 10.3%).

Functional enrichment analyses were then performed on this broader set to explore potential biological pathways associated with the DMRs and DhMRs. Genes associated with DMRs revealed significant enrichment in vascular development and neuronal differentiation pathways. Key genes included *ZMIZ1*, *MYOCD*, *FOXC2*, and *RAPGEF2*, which were associated with processes such as artery morphogenesis, vasculogenesis, and forebrain development (Data S2, Fig. S3). At an FDR < 0.05, genes linked to DhMRs, including *EPHB2, EGFR, KLF4, CLEC7A*, *FOXC2,* and *CDON*, were enriched in pathways related to cell communication, IL-2 production, signal transduction, and neurodevelopment (Data S2).

### Enrichment of D(h)MRs near intron‒exon junctions

**Fig. 3:**
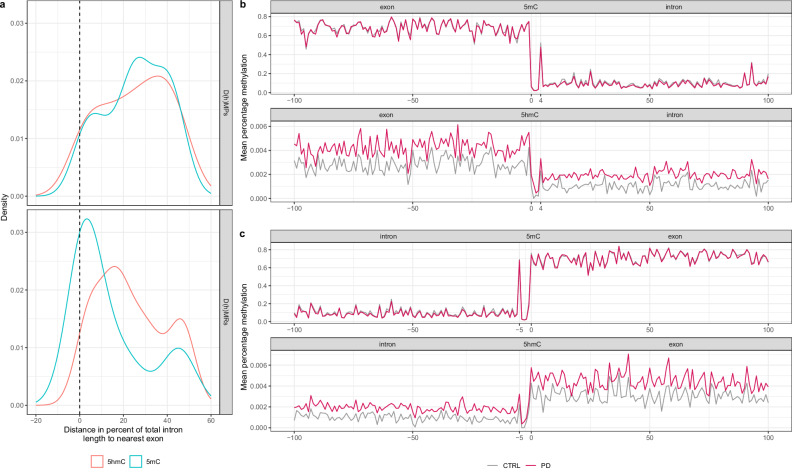
Mapping of probes associated with D(h)MPs and D(h)MRs. **a** Relative spatial distribution of D(h)MPs and D(h)MRs within intronic regions. D(h)MPs display a broadly uniform distribution with a peak near the center of introns, whereas D(h)MRs are more concentrated near intron-exon junctions. 5mC-enriched regions are concentrated within the first 5%, while 5hmC-enriched regions are located within the first 20% of introns. **b** Values represent the mean methylation levels (%) of all 5hmC and 5mC probes located within 100 bp of exon-intron junctions in PD and control (CTRL) groups. The region from −100 to 0 bp represents exonic sequence, and 0 to +100 bp represents intronic sequence. **c** Values represent the mean methylation levels (%) of all 5hmC and 5mC probes located within 100 bp of intron-exon junctions in PD and CTRL groups. The region from −100 to 0 bp represents intronic sequence, and 0 to +100 bp represents exonic sequence.

Building on the observed enrichment of D(h)MPs within intronic regions (Fig. 2c) and the correlation between exon proximity and methylation variability across the whole array (Fig. S2), we hypothesized that intron-exon junctions may play a role in PD pathogenesis. To explore this potential relationship, we mapped all significant intronic D(h)MPs and quantified their relative positions as a percentage distance from the nearest exon–intron boundary. The distribution of these CpG sites across junctional regions is illustrated in Fig. 3a. Probes associated with D(h)MPs displayed a relatively uniform distribution across intronic regions, with a notable peak occurring approximately 20–40% from the intron‒exon junction (Fig. 3a). In contrast, probes within D(h)MRs exhibited pronounced clustering near intron‒exon boundaries. Specifically, 5mC probes from the FWER ≤ 20% list were concentrated within the first 5% of the intron, whereas 5hmC probes were predominantly enriched within the first 20% (Fig. 3a).

Regions located within 0–250 bp of exon–intron junctions were identified for both modifications (DMRs: 3/18, 16.7%; DhMRs: 16/107, 15.0%), based on the distance-to-exon grouping defined in Data S1. However, the majority of DMRs and DhMRs were positioned >500 bp from the nearest exon (DMRs: 14/18, 77.8%; DhMRs: 82/107 76.6%). Thus, in this exploratory dataset, 5mC and 5hmC showed broadly similar positional distributions relative to exons (Data S1). Despite similar distance profiles, the genes associated with proximal and distal regions differed in their functional roles. Proximal DMRs associated with lysosomal and lipid-metabolism genes, such as *ABHD16A* and *TMEM175*, whereas proximal DhMRs were associated with immune and metabolic genes, including *CLEC7A*, *CD5*, *NFKB1*, and *MLXIPL*. Distal DMRs and DhMRs were linked to synaptic, transcriptional, lysosomal, and immune genes (e.g., *TNIK, GM2A, FOXC2, LRRTM4, EPHB2, CDON, LAPTM5, IL34*), consistent with pathways relevant to PD.

To further characterize methylation dynamics at intron‒exon junctions, we mapped all available probes located within 100 bp of either side of the boundary. Comparative analysis of methylation states between PD cases and controls revealed that differences in 5hmC were consistently more statistically significant than differences in 5mC, yielding lower p*-*values at individual CpG sites within these transitional regions (Fig. S4). Moreover, 5hmC levels were generally elevated in PD cases, supported by DhMPs yielding a mean p-value of 0.014 compared to 0.55 for DMPs (Fig. 3b, c). Notably, both 5mC and 5hmC signals exhibited distinct peaks approximately 4 bp downstream of exon ends (Fig. 3b) and 5 bp upstream of exon start sites (Fig. 3c). This pattern suggests a localized sharp spike that may be functionally relevant for alternative splicing. Probe coverage across the 200 bp window depicted in Fig. 3b, c, remained relatively consistent, with approximately 500 probes per nucleotide (Fig. S5). Collectively, these findings highlight a preferential enrichment of methylation and hydroxymethylation at intron-exon junctions in PD, with 5hmC differences showing stronger disease association and generally elevated levels in individuals with PD compared to controls.

### Global 5hmC levels in PBMCs show predictive value for PD status

**Fig. 4:**
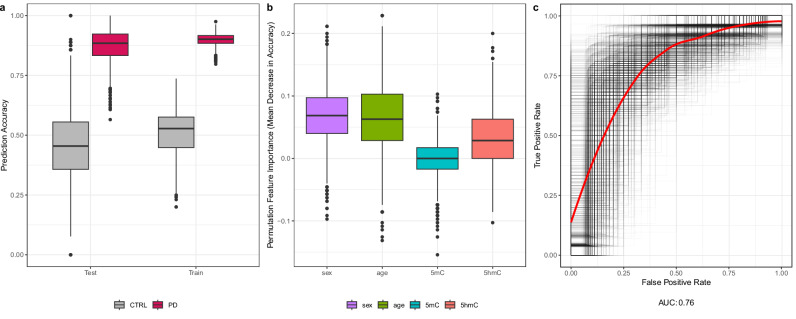
Predictive values of global 5hmC and 5mC levels to determine PD status. **a** Prediction accuracy of the logistic regression model across 2000 random train-test splits, highlighting differences between predicted PD cases and controls (CTRL) subjects. The model demonstrated high accuracy for predicting PD cases (88%), while accuracy was lower for predicting controls (45%) (Training CTRL Mean = 0.53, Training PD Mean = 0.90, Test CTRL Mean = 0.46, Test PD Mean = 0.88). **b** Permutation importance of variables, obtained from test sets using logistic regression modeling, indicating global 5hmC levels as a key predictive variable for PD status (Mean importance: sex = 0.07, age = 0.06, 5mC = 0, 5hmC = 0.03). **c** Receiver operating characteristic (ROC) curves for all train-test-splits (gray), alongside a smoothed ROC curve (red) with AUC = 0.76, demonstrating predictive power of the model.

Given the evidence supporting a functional role for DNA modifications in transcriptional dysregulation in PD, we examined whether global 5hmC and/or 5mC levels could contribute to predicting PD status and disease stage. To this end, we fitted logistic regression models to predict PD status and linear regression models to predict disease stage using available data. To ensure the robustness of our findings, we generated 2000 distinct train-test splits, fitted logistic regression models on the training sets, and recorded the class-based prediction accuracy for PD status in each model. On average, PD cases were predicted with 88% accuracy using global 5mC and 5hmC levels, along with age and sex (Fig. 4a). In contrast, prediction accuracy for controls was lower, at 45% (Fig. 4a). To assess the contribution of each variable, we computed permutation importances^25^ of each variable on the test data across all train-test-splits. Our results indicate that global 5hmC levels were a comparatively strong predictive variable, along with age and sex, whereas global 5mC levels had little predictive value (Fig. 4b). Furthermore, we fitted a receiver operating characteristic (ROC) curve on test data across all 2000 train-test-splits and derived a smoothed representation^26^, yielding an area under the curve (AUC) of 0.76 (Fig. 4c). The role of age, sex, and global 5mC and 5hmC levels in predicting H&Y stage was evaluated using linear regression models over 2000 random train-test splits. The linear model produced a mean absolute error (MAE) of 0.78, with age identified as the only important predictor of disease stage (Fig. S6). Taken together, these findings suggest that previously known factors such as age and sex, together with global 5hmC levels, contribute to predicting PD status, whereas global 5mC levels show minimal influence.

## Discussion

This study offers new insights into PD-related epigenetic alterations by demonstrating global 5hmC hypomethylation in PBMCs and identifying several differentially methylated and hydroxymethylated regions and positions in an exploratory genome-wide analysis, underscoring that 5hmC warrants further assessment and investigation in PD. A recent study similarly reported lower global 5hmC and 5mC in buffy coat samples from individuals with PD compared to controls^21^, supporting the notion that peripheral 5hmC alterations may be a feature of PD. In postmortem PD brain tissue, however, the pattern appears different: studies of the cerebellum have shown elevated global 5hmC levels with no significant changes in 5mC^14,15^, highlighting potential tissue-specific differences in 5hmC regulation. In cortical sections, increased 5mC levels have been observed, whereas global 5hmC levels remained unchanged. Kaut et al. reported unchanged methylation and hydroxymethylation immunoreactivity in the substantia nigra of PD cases compared to controls^15^, while Min et al. identified DhMRs and no DMRs in the same tissue^17^. Overall, these findings differ somewhat from ours, likely reflecting tissue-specific differences. Postmortem brain tissue represents the end stage of PD and may introduce confounding factors related to postmortem physiological and environmental conditions. In contrast, PBMCs have a higher turnover and were collected at different disease stages. Additionally, differences in methodological approaches and quantification platforms may substantially influence outcome measurements. For instance, while our ELISA-based assay revealed global hypohydroxymethylation in PD, genome-wide profiling using Illumina EPIC BeadArrays indicated elevated 5hmC levels at specific CpG sites, particularly within intronic regions. This discrepancy likely reflects differences in genomic coverage and resolution between global and locus-specific assays. Whereas the ELISA captures total 5hmC content across all genomic regions, the genome-wide profiling focuses on gene-associated CpGs, where disease-specific gains in 5hmC may occur despite overall global loss. Importantly, the limited sample size in our study constrains the generalizability of these findings and underscores the need for more comprehensive, large-scale investigations into 5hmC dynamics in PD. Further work in larger PD cohorts will be essential to clarify the biological relevance of these patterns and to validate our findings.

The PLS analyses did not identify significant contributions from factors such as medication, caffeine intake, or exposure to heavy metals and paint/solvents to the global 5hmC hypomethylation observed in individuals with PD within our cohort. A recent study on whole blood from PD cases identified methylation sites influenced by levodopa therapy^27^. Although levodopa’s general effect on one-carbon metabolism could potentially lead to global DNA methylation changes^28^, our PLS analysis did not reveal any significant PC, suggesting a weak or non-existent correlation between levodopa (or other medications) and global 5hmC or 5mC levels. Additionally, when comparing 5mC and 5hmC levels between PD cases on levodopa treatment and those not receiving it, we found no statistically significant difference. However, these results do not rule out the possibility of levodopa and/or other medications affecting specific sites.

The observed changes, including the globally reduced 5hmC levels reported here, may have pathological consequences or may simply reflect downstream effects of the disease itself. Notably, we identified 107 DhMRs compared to only 18 DMRs, a pattern similar to Min et al., who likewise reported DhMRs but no DMRs in substantia nigra tissue from PD cases, although their study detected a larger number of DhMRs overall^17^. In our dataset, the predominance of hypohydroxymethylated DhMRs, together with their genomic localization, suggests that biological factors are likely contributing to the observed pattern. Loss of 5hmC has been reported in PD and other neurodegenerative or stress-related contexts, consistent with alterations in chromatin state, transcriptional activity, or cell-type composition^29^. While technical effects cannot be fully excluded, the overall consistency of the signal supports a biological component. Further investigation will be needed to clarify the implications of global hypohydroxymethylation, as well as the site-specific methylation and hydroxymethylation changes observed across different tissues, and their potential links to PD pathology.

Our enrichment analyses revealed distinct biological patterns associated with 5mC and 5hmC modifications in PD. DMP-associated genes showed enrichment in pathways related to synaptic signaling, cell adhesion, and neurodevelopment, with genes such as *TNIK, GRIK5*, and *EPHB2* implicated in neuronal connectivity and ion channel regulation, suggesting that 5mC alterations may influence synaptic architecture and neuronal communication relevant to PD. DMR-associated genes were enriched in vascular and forebrain development, highlighting genes such as *MYOCD, FOXC2, RAPGEF2*, and *ZMIZ1*. These findings raise the possibility that regional 5mC alterations could affect vascular remodeling and neurodevelopmental trajectories, potentially relevant to disease mechanisms involving neurovascular interactions. In contrast, DhMP- and DhMR-associated genes showed strong enrichment in neurodevelopmental, immune, and signaling-related processes. Key genes included *SMARCA4, HIPK2, EGFR, KLF4, EPHB2*, and *CLEC7A*, associated with transcriptional regulation, interleukin signaling, and cell communication, with *SMARCA4* and *HIPK2* in particular linked to nervous system development. These patterns suggest that 5hmC alterations may influence neurodevelopmental, immune, and signaling pathways relevant to PD pathophysiology.

To deepen this pathway-level interpretation, we examined the genomic positioning of D(h)MRs relative to exon–intron boundaries. A subset of regions occurred within 0–250 bp of exon–intron junctions, where methylation and hydroxymethylation changes could plausibly influence splice-site usage or exon recognition. Proximal DMRs were associated with *KLHL35*, *ABHD16A*, and *CLPSL1*, with *ABHD16A* participating in lysophospholipid metabolism and lysosomal signaling. Proximal DhMRs were associated with immune and metabolic regulators such as *CLEC7A*, *CD5*, *NFKB1*, and *MLXIPL*, placing several microglial and metabolic genes in splice-proximal contexts. These positional patterns raise the possibility that PD-associated epigenetic variation may influence splicing-related regulatory mechanisms.

Several additional genes emerging from DMPs, DMRs, DhMPs, and DhMRs have established roles in neuronal vulnerability. Neurotransmission-related loci such as *GRIK5*^30^ and *KCNQ1*^31^ regulate glutamatergic signaling and neuronal excitability, both disrupted in PD. Synaptic regulators, including *TNIK*, *BAIAP2*, *EPHB2*, and *EGFR,* influence MAPK signaling, actin dynamics, and neurotrophic support, with recent work implicating *TNIK* in postsynaptic density function^32^ and *EGFR* in dopaminergic neuron maintenance^33^, and with EGFR expression reported to be downregulated in PD brain tissue^34^. Chromatin and stress-response genes (*SMARCA4*, *HIPK2*, *ZHX2*) have been implicated in pathways relevant to PD. *SMARCA4* influences dopaminergic neuron survival and shows neuroprotective effects when inhibited in experimental models^35^, while *HIPK2* supports mitochondrial quality control through Parkin-dependent mitophagy and is reduced in PD brain tissue^36,37^. *ZHX2*, a transcription factor involved in neuronal differentiation, has shown nominal associations with cognitive impairment in PD, suggesting a role in transcriptional dysregulation^38^. Neurodevelopmental genes such as *AHI1*, involved in cortical development and vesicle trafficking and upregulated in Gba-mutant mice^39^, together with *OLFM1*, a regulator of neuronal development and synapse structure that is reduced in PD brain tissue^40^, suggest that altered developmental pathways may confer lasting susceptibility to degeneration. Vascular and immune-related genes (*FOXC2*, *MYOCD*, *CLEC7A*) further point toward cerebrovascular and microglial contributions to PD, consistent with evidence of vascular degeneration and innate immune activation in the disease^41,42^. *CLEC7A*, encoding the microglial pattern-recognition receptor Dectin-1, can modulate microglial responses in PD-related contexts^43^. Together, these gene-level patterns, considered alongside the positional enrichment of D(h)MRs near exon–intron boundaries, provide a mechanistic framework linking 5mC and 5hmC variation to synaptic, immune, metabolic, and splicing-related processes relevant to PD.

Overall, our findings point to a layered epigenetic architecture in PD, where 5mC alterations may affect structural and vascular pathways, while 5hmC marks appear to engage more specific regulatory programs involved in neuronal development, immune modulation, and intercellular signaling. Although these enrichments were identified in PBMCs and may not fully reflect the epigenetic landscape of disease-relevant brain regions, they may capture systemic regulatory signatures or shared gene expression profiles. Together, these patterns provide insight into how distinct DNA methylation marks could contribute to PD pathogenesis and progression through complementary, context-dependent mechanisms.

Analysis of genome-wide 5mC and 5hmC patterns revealed that D(h)MPs were enriched in intronic regions and depleted in intergenic regions and CpG islands, suggesting a tendency toward gene-body rather than intergenic or promoter-associated targeting. D(h)MRs also showed a localized enrichment near intron-exon junctions. The presence of both 5mC and 5hmC changes in these junctional regions suggests potential effects on splicing-related regulatory processes. Prior studies have reported elevated DNA methylation levels in exons relative to flanking introns, particularly at splice sites, where methylation has been proposed to influence splicing by interacting with the splicing machinery and facilitating the recognition of alternative exons^44–48^. Tissue-specific DMRs have also been frequently observed in first introns^49^. A potential role for 5hmC in RNA splicing and synaptic function has been suggested as well. Khare et al. identified 5hmC enrichment in synaptic genes within the human frontal cortex and reported higher 5hmC levels in constitutive exons compared to alternatively spliced exons^50^. Additional studies not conducted in the context of PD have indicated that 5hmC may influence gene expression when located near transcription factor binding motifs and may modulate alternative splicing when positioned close to intron‒exon boundaries^51,52^.

These spatial patterns of epigenetic modification raise questions about their functional relevance in PD. Several PD-associated genes, including *SNCA*, *LRRK2*, and *PRKN* (formerly known as *PARK2)*, undergo alternative splicing, and dysregulation of isoform expression has been linked to PD pathology^53^. The enrichment of D(h)MRs near intron–exon junctions therefore raises the possibility that epigenetic modifications could influence splice-site selection or exon inclusion in these genes. Additionally, intronic 5hmC has been associated with enhancer activity and transcriptional elongation^54,55^, suggesting that the modifications identified here might affect gene expression kinetics or isoform balance^56^. Together, these findings, in combination with prior evidence, suggest that intronic enrichment of D(h)MPs and D(h)MRs may reflect epigenetic modulation of splicing and transcriptional regulation within gene bodies. Such mechanisms could contribute to altered isoform usage or gene expression dynamics in disease-relevant pathways, although functional studies will be required to test these possibilities.

Identification and implementation of biomarkers are essential for early and accurate diagnosis, monitoring disease progression, and assessing therapeutic responses in PD. Biomarkers detectable in peripheral tissues, such as blood, offer practical advantages, and blood-based markers of neurodegeneration are emerging as promising tools^57^. In this study, we found that PD status could be predicted with moderate accuracy using global 5hmC levels in combination with age and sex, with 5hmC emerging as a comparatively influential predictive variable. One limitation of this model is the skewed sex distribution in the control group, which may have inflated the predictive contribution of sex and influenced overall model performance. Additionally, we were unable to reliably distinguish between disease stages using the same parameters, likely reflecting limited sample size and/or substantial interindividual variability. Larger cohorts will be needed to determine whether global 5hmC levels can predict disease stage and to further evaluate the potential links between DNA modifications and alternative splicing in PD. Altered 5hmC levels have previously been linked to aging and neurodegenerative disorders^7,58^. Our findings suggest that 5hmC levels may hold promise as a peripheral biomarker for early detection or longitudinal monitoring. Nonetheless, validation in larger and more diverse cohorts will be essential.

This study has several limitations that should be considered when interpreting the findings. Although our genome-wide analysis identified D(h)MRs enriched at exon–intron junctions and suggested reduced global 5hmC levels in PD, these results remain preliminary. Independent validation using quantitative biochemical assays and targeted sequencing approaches in larger cohorts will be essential to confirm these observations. First, the exploratory cohort was small, which limits statistical power and increases the likelihood of both false negatives and inflated effect-size estimates among detected loci and regions. Consequently, the large Δβ values observed in some D(h)MPs and D(h)MRs should be interpreted cautiously, as they may partially reflect sampling variability rather than true biological magnitude. Second, although multiple-testing correction was applied, the large number of CpG sites relative to the small sample size means that some identified D(h)MPs and D(h)MRs may represent false positives. Third, PBMC-based methylation profiles may be influenced by lifestyle or immune-related factors. In our dataset, smoking status, caffeine intake, pesticide exposure, and medication use did not show measurable effects in PLS analyses, and flow-cytometry data from a subset of participants showed no major differences in PBMC cell-type distributions. Although this subset only partially overlapped with the samples used for global 5mC/5hmC profiling and included two controls from the genome-wide analysis, all participants were drawn from the same cohort, making substantial differences in cell composition unlikely. Nonetheless, some residual confounding by cell-type distribution cannot be entirely excluded. Finally, the global 5mC and 5hmC measurements were cross-sectional, providing only a single time point per participant. This limits our ability to determine when these epigenetic differences arise or how they change over the course of PD, and larger cohorts will be needed to validate these findings. In contrast, the genome-wide methylation analysis was restricted to individuals with H&Y stage 4 compared to controls, which prevents assessment of methylation changes across earlier disease stages. These limitations underscore the importance of validating these findings in larger, independent cohorts and pursuing functional studies to determine the biological relevance and potential clinical utility of the identified epigenetic signatures.

In conclusion, our findings indicate that individuals with PD exhibit global 5hmC hypomethylation in PBMCs, along with several D(h)MRs and D(h)MPs identified in a small exploratory subset subjected to genome‑wide analysis. Global 5hmC levels measured in blood showed potential to distinguish PD cases from controls, suggesting that they may warrant further investigation as a biomarker. The enrichment of D(h)MPs near intron–exon boundaries is an intriguing observation. However, the functional consequences of these locus‑specific changes remain unknown. Some of the affected regions map to genes involved in diverse biological themes, raising the possibility that 5hmC alterations may relate to pathways relevant to PD biology. Future studies with larger cohorts and orthogonal validation methods will be required to determine whether these epigenetic alterations influence RNA processing, transcriptional regulation, or other molecular pathways relevant to PD.

## Methods

### Participants and clinical data

This study was conducted in accordance with the Declaration of Helsinki and Good Clinical Practice guidelines after approval of the local ethical committee. Informed consent was obtained from all study participants. A total of 109 individuals with idiopathic PD and 49 neurologically healthy controls were recruited from Karolinska University Hospital (Fig. S7). Genetic status for *LRRK2*, *SNCA,* and *GBA* mutations was determined previously by genotyping^23,59^. No *LRRK2* or *SNCA* mutations were detected. PD cases carrying GBA mutations encoding N370S (*n* = 3) and L444P (*n* = 2) were excluded prior to analysis and are not included in the 109 cases. The E326K mutation was detected in nine PD cases, who were retained in the cohort. A subanalysis was performed excluding these carriers. PD cases had been diagnosed between 36 and 86 years of age. At blood collection, participants were 38-87 years old in the PD group and 41–78 years old in the control group. Controls were recruited and included spouses, unaffected family members, and unrelated individuals. Relevant clinical data, including information such as medication intake, caffeine intake, exposure to solvents/heavy metals, and smoking, were collected upon written informed consent. The H&Y scale was employed to evaluate disease stage in the PD cohort. Disease duration was calculated as the number of years between clinical diagnosis and the blood sampling used for PBMC collection. Ethical approval was obtained from the Regional Ethical Review Board in Stockholm (Dnr: 2011/500-31/1; 2013/19-32). Patient material was collected at Karolinska University Hospital, Sweden, after written informed consent and stored in the local biobank at the Centre for Molecular Medicine (CMM), Karolinska Institutet, Sweden.

### Blood collection and PBMC isolation

Blood samples were collected in EDTA tubes by venipuncture from individuals with PD and controls. PBMCs were isolated by low-density gradient centrifugation using Lymphoprep (Axis-Shield) and subsequently stored at −80 °C.

### DNA extraction from PBMCs

DNA was extracted from PBMCs using the PureLink Genomic DNA Mini kit (Invitrogen) with an on-column purification protocol following the manufacturer’s instructions. Total DNA was quantified using the Qubit dsDNA BR assay on a Qubit Fluorometer (ThermoScientific). Extracted DNA was stored at +4 °C until use.

### Quantification of global DNA methylation and hydroxymethylation levels

The extracted DNA was used to quantify global levels of DNA methylation and hydroxymethylation using the MethylFlash Methylated DNA Quantification Kit (Epigentek, P-1035) and the MethylFlash Hydroxymethylated DNA Quantification Kit (Epigentek, P-1037) following the manufacturer’s instructions. For each sample, 100 ng of input DNA was used for 5mC measurements and 200 ng for 5hmC measurements. The kits provided highly specific capture antibodies for methylcytosine and hydroxymethylcytosine, with no cross-reactivity to unmethylated cytosine or to the alternate modified base. Negative and positive DNA controls supplied with the kits were included in all assays. For relative quantification, 5 ng of positive control was used for 5mC and 2 ng for 5hmC. Detection was performed according to the manufacturer’s instructions. Briefly, genomic DNA was added to the assay wells, followed by careful washing and addition of the capture antibody. After a second wash, the detection antibody and enhancer solution were added. The capture antibodies bind specifically to 5mC or 5hmC. Fluoro-development solution was then added, and relative fluorescence units (RFU) were measured at 530ex/590em using a GloMax®-Multi+ Microplate Multimode Reader (Promega). Fluorescence intensity is proportional to the amount of hydroxymethylated or methylated DNA in the sample. Relative quantification was used to determine global methylation and hydroxymethylation levels. Percent 5mC or 5hmC in total DNA was calculated using the following formula: 5mC % or 5hmC % = (((Sample RFU–negative control RFU) ÷ S)/((positive control RFU—negative control RFU) × (2* ÷ P))) × 100% where RFU = relative fluorescence units; S = input sample DNA (ng) and P = input positive control DNA (ng). Samples showing poor replicate concordance (>250 RFU difference between duplicates) were excluded from the analysis (5mC: nine PD and three control samples; 5hmC: one PD and three control samples).

### DNA methylation and hydroxymethylation analysis using the MethylationEPIC v1.0 BeadChip

Genome-wide methylation and hydroxymethylation profiling was performed on DNA from eleven biological samples (PD cases *n* = 6; controls *n* = 5). Each sample underwent both bisulfite and oxidative bisulfite conversion, yielding a total of 22 converted DNA aliquots (Fig. S7). Conversions were carried out on 1000 ng of DNA per sample using the TrueMethyl® Kit (Cambridge EpiGenetix), according to the manufacturer’s protocol as described in the TrueMethyl® Seq Kit User Guide (version 3.1). Interrogation of DNA oxidation and qualitative assessment of 5-hmC oxidation and bisulfite conversion were performed according to the protocol and confirmed for all samples. The PD and control samples were age- and sex-matched, except for PD-1 and C-1, which were age-matched but not sex-matched (see Table S2). Genome-wide methylation profiling of the 22 converted DNA samples was performed using 7 µl of the recovered TrueMethyl template from each sample, combined with 1 µl 0.4 NaOH, and processed on the Illumina MethylationEPIC_v1-0 DNA analysis BeadChip (Illumina Inc.) according to the manufacturer’s protocol. The probe call rate at a detection p-value of 0.01 exceeded 98% for all samples, except for control sample 2 (C-2) (Table S2), which showed a rate of 94.8% and was excluded from the final analysis.

Data were initially preprocessed using the minfi R package. Quality control procedures and exploratory analyses performed with the package’s built-in functions confirmed that the data were suitable for subsequent analyses. To estimate 5mC and 5hmC intensities from the available data, we applied the oxBS-MLE method^60^. This approach uses a maximum likelihood estimation algorithm to derive the corresponding beta values. The resulting datasets underwent additional quality control to confirm their suitability for analysis. All samples, except C-2, were included in downstream analyses (Fig. S7).

### Identification of D(h)MPs and D(h)MRs

To identify D(h)MPs, we used the dmpFinder function in the minfi R package. Each methylation type was subjected to the dmpFinder function to identify those positions that differed between disease states. Significance was determined by selecting probes with an FDR below 10%. To expand on the D(h)MPs and identify potential D(h)MRs, we applied the approach within the bumphunter R package using the same model matrix as for dmpFinder. Here, the genomic distribution of CpGs measured on the Illumina EPIC BeadArray is subset into more distinct regions based on genomic distance. To assess whether the identified regions differed significantly from what would be expected by chance, we applied the built-in bootstrapping procedure to compute FWER. The primary analysis tested region-level differences (D(h)MRs) while controlling the FWER at 5%, with secondary exploratory analyses conducted at FWER ≤ 20% to broaden hypothesis generation. For all results, we report adjusted p-values, associated statistics, and effect sizes, as well as the direction of change of adjacent probes within a +/-2500 bp window.

Leukocyte composition was previously quantified by flow cytometry in the same PBMC cohort from which the subset was drawn, and no significant differences in major immune cell populations were observed between PD and control samples^22^. Therefore, no additional adjustment for cell-type proportions was applied in the genome-wide analyses. Genomic locations used within the analysis were extracted from the TxDb.Hsapiens.UCSC.hg19.knownGene R package. Additional information on CpG islands was extracted from the latest Illumina BeadChip Annotation package (v 1.0 B4). To identify whether a genomic location or CpG island was significantly enriched, we used a bootstrapping approach. Here, we randomly selected 1000 subsets of probes of equal size to the differentially expressed positions and calculated their respective proportions. These proportions were then used as input to an empirical cumulative distribution function to calculate the probability of the given genomic location being enriched or depleted. An FDR < 10% cutoff was used to define significance.

### Defining the methylation state in relation to intron‒exon junctions

To analyze methylation states in relation to intron‒exon junctions across the genome, we first identified all known intron locations and mapped available methylation features to these regions. Since intron lengths vary, we standardized CpG positions within introns based on their relative distance to the nearest exon. For simplicity, we defined the midpoint of each intron as 50% of the distance between its flanking exons. Consequently, each intron-mapped CpG was assigned a value between 0 and 50%. We then visualized the distribution of these CpGs to identify clustering patterns of significant methylation sites within introns. Additionally, we mapped CpGs located within 100 bp of intron‒exon junctions, considering both transitions from intron to exon and exon to intron, and calculated the median beta for each position. *P* values are calculated by comparing all available beta values per position using a Wilcoxon signed-rank test. *P* values across positions were then summarized by taking the geometric mean across the 200 bp region.

### Distance to exon-grouping

For the distance-to-exon assessment, minimal distances between CpGs within each D(h)MR and the nearest annotated exon–intron boundary were calculated based on hg19 gene annotations. D(h)MRs were then assigned to distance categories defined as proximal (0–250 bp), intermediate (250–500 bp), or distal (>500 bp), using the minimum CpG-exon distance per region.

### Associations between PD and global DNA methylation and hydroxymethylation levels

Associations between levels of 5mC and 5hmC within PD were tested using multivariate linear regression, with 5mC and 5hmC levels as outcomes and PD as exposure, controlling for age and sex as possible confounding variables (RStudio). Acceptable levels of Gaussianity in the residuals were verified using QQ plots. Multivariate modeling was performed using Simca (version 15.0.0, Sartorius Stedim Biotech, https://umetrics.com) with all variables scaled to unit variance. When calculating orthogonal components, 7-fold cross-validation was applied, and components with *Q*^2^ > 0.05 were considered significant PCs. By default, the regression coefficients correspond to the centered and scaled data and were derived from all significant components. Coefficients were displayed with a confidence interval computed by jack-knifing. VIP scores were calculated for each x_k_ by summing the squares of the PLS loading weights (wa_k_), weighted by the proportion of the sum of squares explained in each model component. The sum of squares of all VIP’s is equal to the number of terms in the model. The VIP score reflects the importance of terms in the model, both with respect to Y, i.e., correlation to all the responses, and with respect to X (the projection). VIP is normalized such that the average squared VIP value equals 1. A threshold of a VIP score ≥1.0 was used to identify discriminatory variables. Non-continuous variables were scored as follows: Smoking status 1: categorizes the subject based on if the person is smoking regularly at the time of blood sampling. Former smokers were categorized as non-smokers (former/never smoker = 0; current smoker = 1); Smoking status 2: categorizes the subject into three categories based on their smoking habits at the time of blood sampling (never smoker = 0; current smoker = 1; former smoker = 2). Former smokers reported having smoked previously and were not smoking at the time of blood collection. All former smokers in the PD group stopped smoking >7 years prior to the blood sampling date. The number of cigarettes a person smoked per day was not taken into consideration. Social smokers were categorized as “never smokers” in Smoking status 1 and excluded from Smoking status 2 (*n* = 3). Individuals with PD were asked to report their exposure to pesticides, heavy metals, or paint/solvents at the time of blood sampling. The question was only related to if they have been exposed through their occupation (never exposed = 0; currently exposed/previously exposed = 1). All exposures in the cohort were self-reported.

### Functional enrichment analysis

Functional enrichment of genes associated with significant positions and regions (DMPs, DhMPs, DMRs, and DhMRs) was performed using the gprofiler2 package in R^61^. No formal enrichment analysis was performed for the distance-to-exon grouping assessment. Functional themes for this analysis were assigned through manual curation based on known gene functions and established biological roles described in the published literature (e.g., lysosomal, lipid metabolism, immune signaling, synaptic regulation).

### Predictive modeling of disease status

To establish the predictive power of global 5mC and 5hmC measurements for disease status, we used a modeling approach based on logistic regression. We created 2000 75–25% random train-test splits of our cohort and fitted a model on each training set using age, sex, global 5mC, and global 5hmC levels as variables. 75% of the data is thus randomly assigned to the training data and the remaining 25% to the test data. For each fitted model, class-based prediction accuracies and permutation importances were recorded, and a ROC curve was developed. Both permutation importances and ROC curves were obtained on the test sets. We summarized the prediction accuracies and variable importance across all train-test splits using boxplots and summarized the ROC curves in a single plot containing each individual ROC curve and a LOESS smoothed ROC curve. The AUC of the averaged curve was recorded as a representation of overall predictive power.

## Supplementary information

Supplementary Information

Data S1

Data S2

## Data Availability

Derived data supporting the findings of this study are available from the corresponding author, K.B., upon reasonable request.

## Code availability

All code used for data analysis is available from the corresponding author upon reasonable request.
