# Supplementary material for: Profiling of 5-hydroxymethylcytosine in blood reveals preferential enrichment at exon-intron junctions and predictive value for Parkinson’s disease
Source: NPJ Parkinsons Dis. 2026 Mar 20;12:76. doi: 10.1038/s41531-026-01322-x (PMC13022309; doi:10.1038/s41531-026-01322-x)
Supplement: Supplementary file 1 — Supplementary Information [file 41531_2026_1322_MOESM1_ESM.pdf]

Supplementary Information

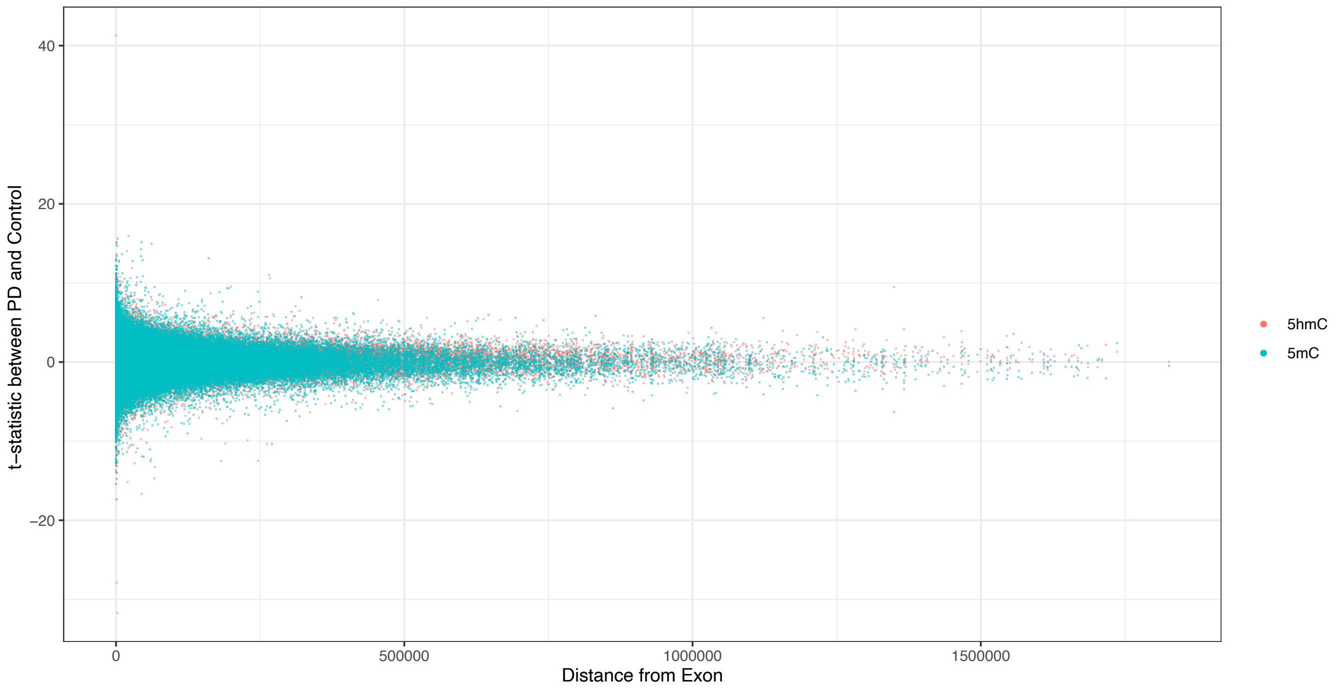

Supplementary Figure 1 Relationship Between Exon Proximity and Differential (Hydroxy)Methylation in Parkinson's Disease

Relationship between the distance to the nearest exon and the degree of differential (hydroxy)methylation in the dataset (PD versus controls), as measured by t-statistics. CpG sites located closer to exons show a higher likelihood of differential methylation, reflected by increasing absolute t-statistic values. A positive t-statistic indicates increased average methylation in PD compared to controls at that CpG site, whereas a negative t-statistic indicates decreased average methylation in PD compared to controls.

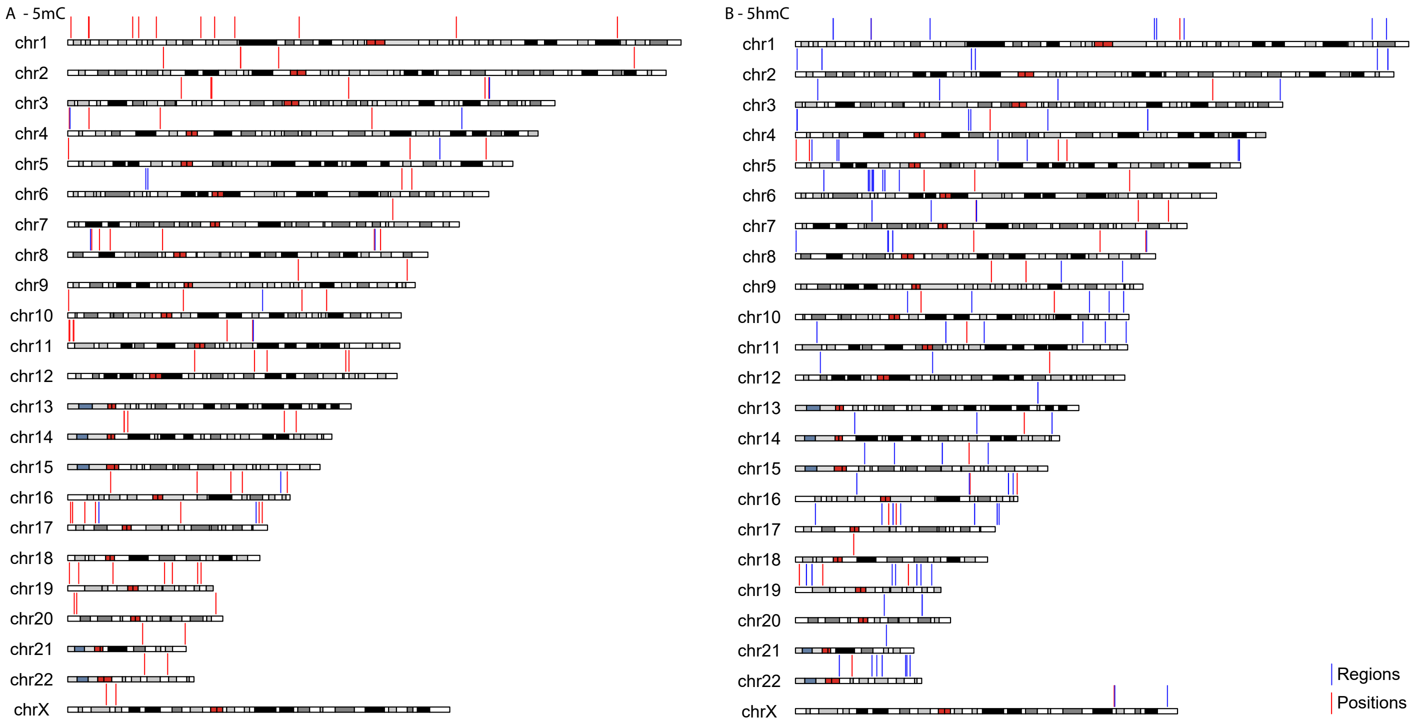

Supplementary Figure 2 Chromosomal Distribution of D(h)MPs and D(h)MRs
Mapping of the D(h)MPs (red) and D(h)MRs (blue) in the (A) 5mC and (B) 5hmC datasets across the chromosomal landscape. No specific chromosomal enrichment was observed for either differentially (hydroxy)methylated positions or regions.

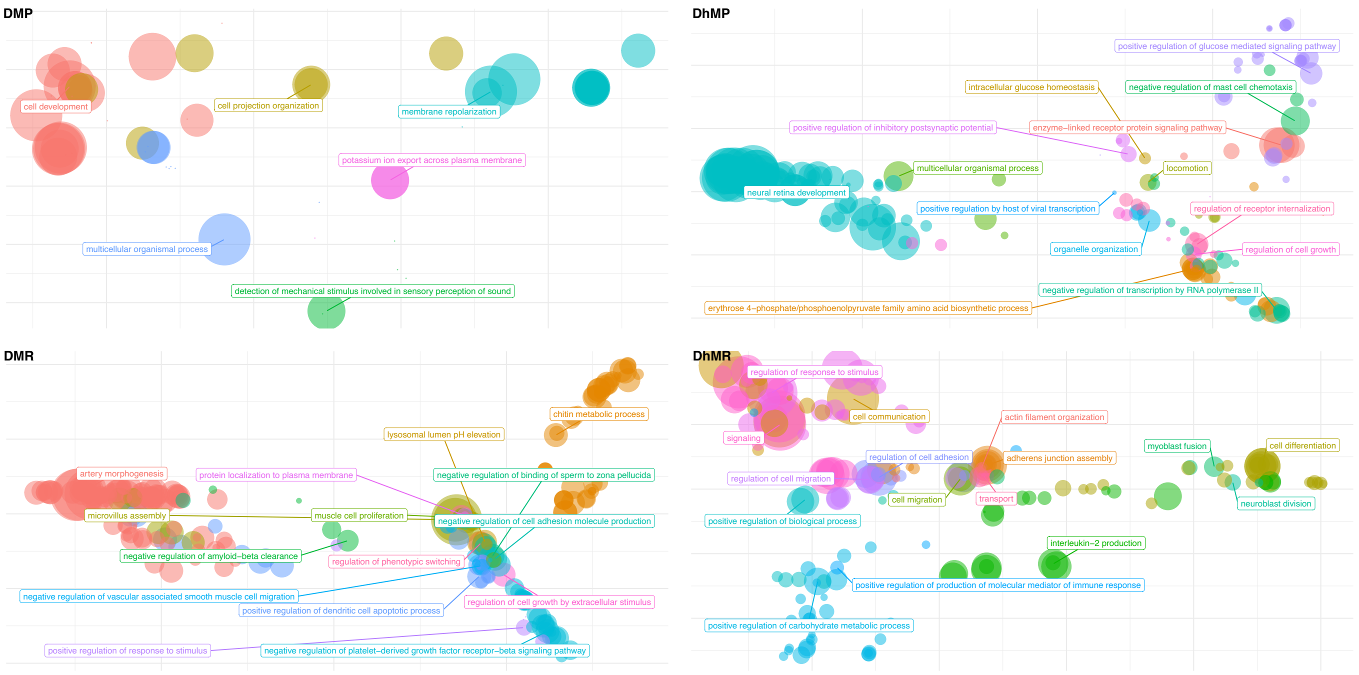

Supplementary Figure 3 GO Term Enrichment Analysis of D(h)MPs and D(h)MRs
Functional enrichment of Gene Ontology (GO) terms associated with D(h)MPs and D(h)MRs was summarized using the R package rrvgo to highlight parental terms. Each circle represents a significantly enriched Biological Process GO term, with colors indicating shared parental terms (labelled accordingly). GO-terms with the same colour share the same parental term which is labelled. Circle size reflects the significance of enrichment, scaled by -log10(p-value) scale. The x- and y-axes denote semantic distance between GO terms such that circles positioned closer together share more genes than circles farther apart.

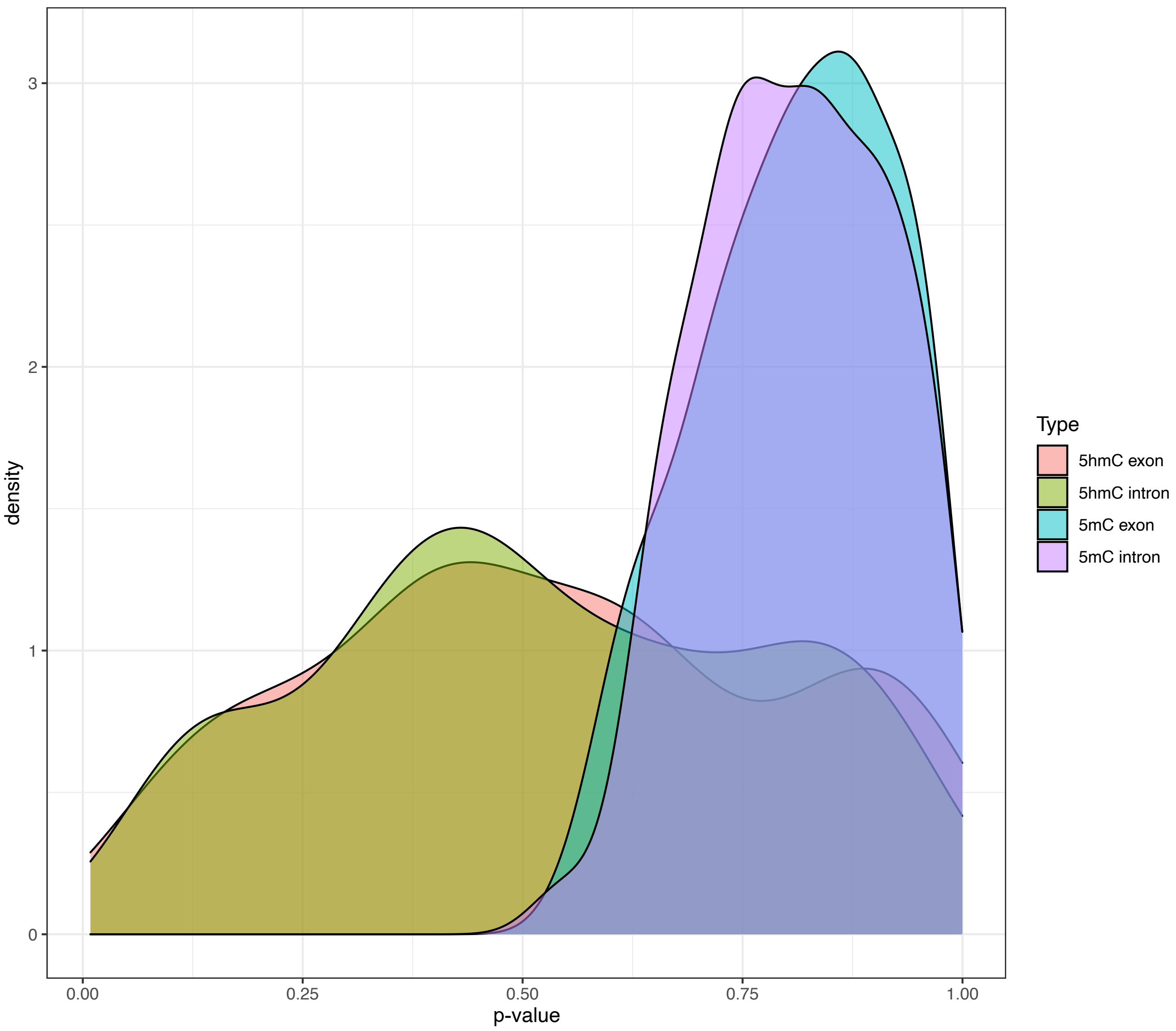

Supplementary Figure 4 Distinct P-value Profiles of 5hmC versus 5mC Probes Around Intron-Exon Junctions
P value distributions comparing PD and controls across the 100 bp flanking intron–exon junctions. 5hmC probes generally exhibited lower P-values and a more uniform distribution compared to 5mC probes.

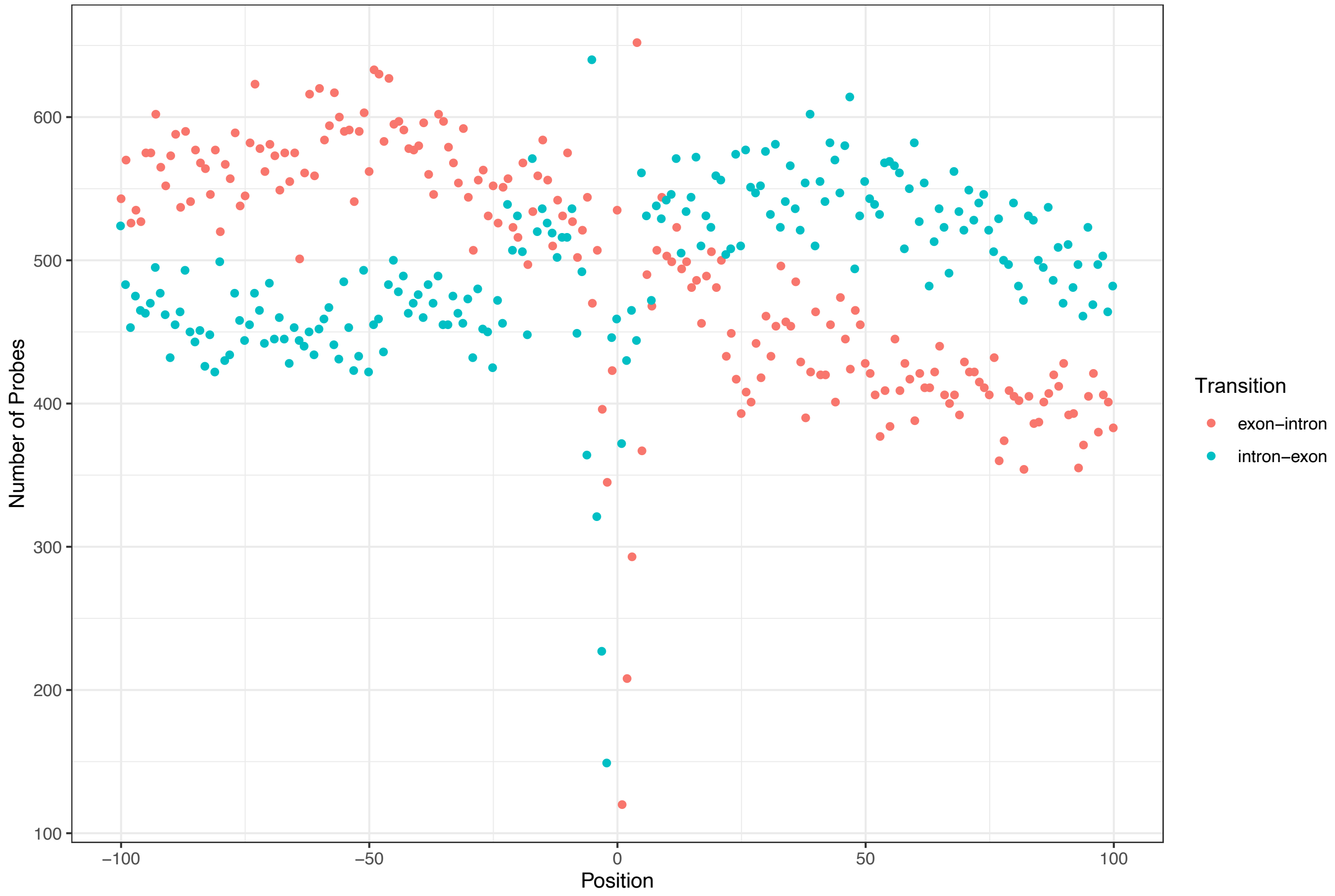

Supplementary Figure 5 Probe Distribution Across Intron-Exon Junctions

Number of probes identified within 100 bp of an intron–exon junction. On average, ~500 probes are present on the EPIC array, irrespective of intron side. A reduction in CpG density was observed only at the junction itself, between positions -2 and +2. More specifically, within the first 2 bp of the intron–exon boundary (position 0), probe counts dropped but still retained approximately 200 probes.

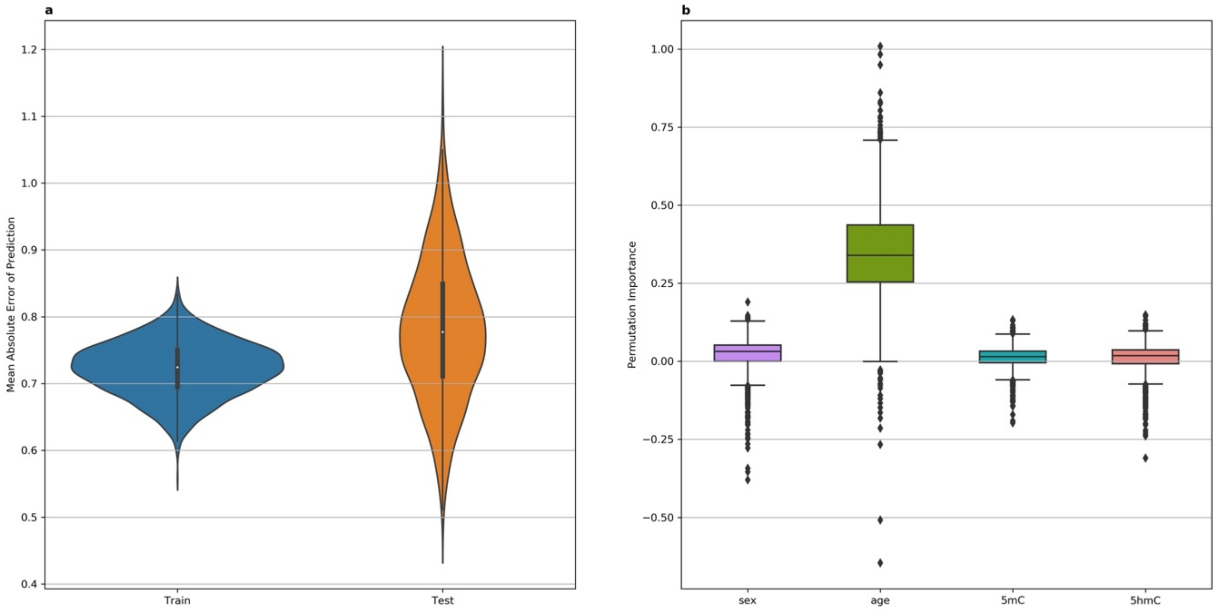

Supplementary Figure 6 Predictive Value of Sex, Age and Global 5hmC and 5mC Levels for PD Progression
Predictive value of sex, age and global 5hmC and 5mC levels for PD progression as measured by Hoehn&Yahr (H&Y). (a) Mean absolute error (MAE) for H&Y prediction using linear regression, with a median MAE of 0.78. (b) Permutation importances of variables, derived from test sets using linear regression modeling, identified age as the only significant predictive variable.

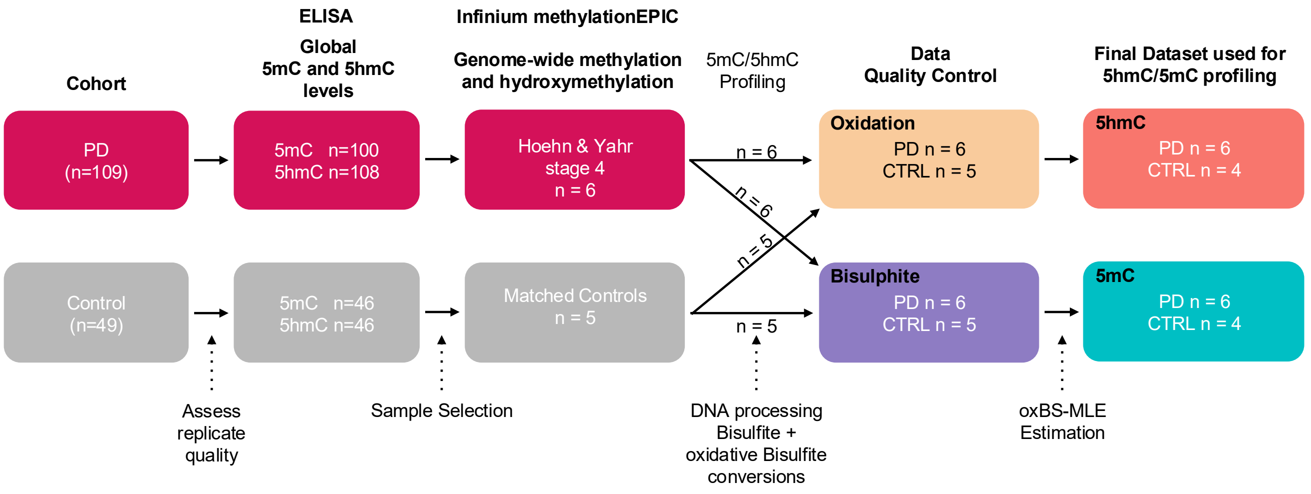

Supplementary Figure 7 Workflow of the study

Supplementary Table 1

Parkinson medications used by PD patients in cohort

PD specific medication	Number of patients on medication, n (%)	Class of drug
Adenosine A2A antagonist	3 (1.6%)	Adenosine A2A antagonist
Amantadine	5 (2.7%)	NMDA antagonist
Apomorphine	2 (1.1%)	DA agonist
Rasagiline	13 (7.1%)	MAOB inhibitor
Entacapone	2 (1.1%)	COMT inhibitor
Levodopa/ benserazide/ carbidopa	80 (43.7%)	Levodopa
Rotigotine	3 (1.6%)	DA agonist
Trihexyphenidyl	6 (3.3%)	Antimuscarinic
Ropinirole	23 (12.6%)	DA agonist
Selegiline	6 (3.3%)	MAOB inhibitor
Carbidopa/ Entacapone	17 (9.3%)	Decarboxylase/ COMT inhibitors
Pramipexole	23 (12.6%)	DA agonist

Supplementary Table 2

Demographic and clinical characteristics of subjects analyzed using the Illumina Beadarray

Subject	Sex[1]	Age[2]	Disease Duration[3]	Hoehn&Yahr stage[4]	PD medication[6]
PD-1	M	70	8	4	Madopark
C-1	F	70		0	
PD-2	M	73	18	4	Madopark, Stalevo, Apo-Go, Sifrol, Amantadine
C-2[5]	M	73		0	
PD-3	F	75	4	4	Madopark
C-3	F	75		0	
PD-4	M	78	16	4	Madopark, Stalevo, Sinemet, Sifrol,
C-4	M	78		0	
PD-5	F	76	10	4	Madopark, Stalevo, Levocar, Requip
C-5	F	76		0	
PD-6	F	76	0	4	Madopark

[1]Male=M; Female=F
[2]Age at blood sampling/PBMC isolation
[3]Years between clinical PD diagnosis and blood sampling/PBMC isolation
[4]Hoehn&Yahr stage at blood sampling/PBMC isolation
[5]Probe call rate at p-detection = 0.01 was 94.8% for this sample; therefore, it was excluded from the final analysis.
[6]PD medication intake at the time of blood sampling/PBMC isolation.

Data S1

This Excel file includes four tables, each located on a separate sheet.

Tables DMPs and DhMPs:
Identified significant differentially (hydroxy)methylated positions (D(h)MPs) between PD and CTRL. *Exondistance* shows the distance to the nearest exon (in bp). A value of 0 indicates that the probe lies anywhere within an exon of the annotated gene. *log(Δbeta)* represents the difference in methylation between PD and CTRL. For each significant probe, we additionally report the number of probes located within ±2500 bp and summarize their direction of change in two columns: *Negative T-Statistic* (%) indicating the percentage of nearby probes showing decreased methylation, and *Positive T-Statistic (%)*, indicating the percentage showing increased methylation. Agreement between the sign of the T-Statistic and the two percentage columns provides additional confidence in the observed significance.

Tables DMRs and DhMRs:
Identified significant differentially (hydroxy)methylated regions (D(h)MRs) between PD and CTRL.
Difference in Area denotes whether the region is hyper- or hypomethylated. Raw p-values are reported, and statistical significance is determined using the family-wise error rate (FWER).
Distance from gene refers to the number of base pairs between the D(h)MR and the nearest gene boundary (start or end). A value of 0 indicates that the region lies anywhere within the gene body, from the transcription start site to the end of the longest annotated transcript.
As for DMPs and DhMPs, we summarize all probes located within ±2500 bp of each D(h)MR and report the proportion showing negative T-statistics (*Negative T-Statistic* %), positive T-statistics (*Positive T-Statistic %)* and no valid T-statistic *(No value %)*. We additionally provide the minimum, mean, and maximum distances of probes within each region to the nearest exon based on hg19 gene annotations.
Distance group (Proximal/Distal): Regions were stratified according to their minimum distance to the nearest exon**,** reflecting the closest potential regulatory interaction point between the D(h)MR and the nearest exon boundary. Regions within **0–250 bp** were classified as **proximal,** representing the splice-site regulatory zone; regions **250–500 bp** were classified as **intermediate,** representing the chromatin–splicing coupling zone; and regions **>500 bp** were classified as **distal,** representing enhancer-associated and gene-body regulatory regions.

Data S2

GO-Term enrichment for the four lists of significant positions and regions (DMPs, DhMPs, DMRs, and DhMRs) identified in Data S1. The Excel file contains four separate tables, one for each list. Results include GO Biological Processes, Cellular Components, and Molecular Function, along with the associated gene names and Ensemble gene IDs.
